# 321. Clinical Utility of Metagenomic Next-Generation Sequencing in Fever of Unclear Etiology: a Single Center Retrospective Cohort Study

**DOI:** 10.1093/ofid/ofac492.399

**Published:** 2022-12-15

**Authors:** Marilyne Daher, Roumen Iordanov, Ahmed M Hamdi

**Affiliations:** Baylor College of Medicine, Houston, Texas; Baylor College of Medicine, Houston, Texas; Baylor College of Medicine, Houston, Texas

## Abstract

**Background:**

Metagenomic next-generation sequencing (mNGS) has emerged as a novel diagnostic tool in infectious diseases. It analyzes genetic material from a given sample without the need for pre-determined sequences, matching it to reference genomes, thus identifying a wide range of pathogens. There is little guidance on the efficient and appropriate use of this tool. The goal of this study was to evaluate clinical utility of mNGS in patients with fever of unclear etiology.

**Methods:**

We retrospectively analyzed 72 patients at our center from June 2017 to July 2021 with “fever of unclear etiology” and a mNGS test. The assay we used was the Karius Test™, a College of American Pathologists-accredited NGS laboratory. Two independent reviews of each case were performed to determine clinical impact of mNGS using previously published criteria by Hogan et al. Logistic regression was used to identify factors associated with positive clinical impact.

**Results:**

We included 72 patients, 62.5% males, median age 56. Most common comorbidities were hypertension (43.1%) and diabetes (23.6%). All patients had fever at time of evaluation. 26.4% had cough, 16.7% abdominal pain, and 15.3% shortness of breath. Median turnaround time of the test was 26 hours. At least one organism was reported in 65.3% of cases. Most common identified organisms were EBV (13.9%), CMV (12.5%), and Rickettsia typhi (11.1%). Of those with an infectious etiology of their fever, 86.8% had positive mNGS compared to 43.8% of those with non-infectious etiology (p=0.002). mNGS had positive clinical impact in 40.3% of cases, negative impact in 2.8%, and no impact in 56.9%. Based on logistic regression, older age was the only factor associated with higher odds of positive impact. Chief complaint, positivity of cultures, number of pathogens retrieved, and type of prior antibiotic use were not associated with a higher rate of positive impact.

**Table 1:**
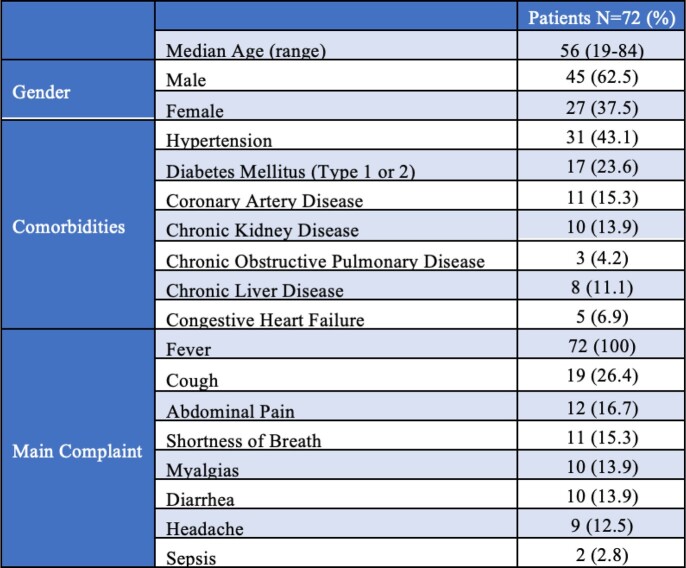
Demographics

**Table 2:**
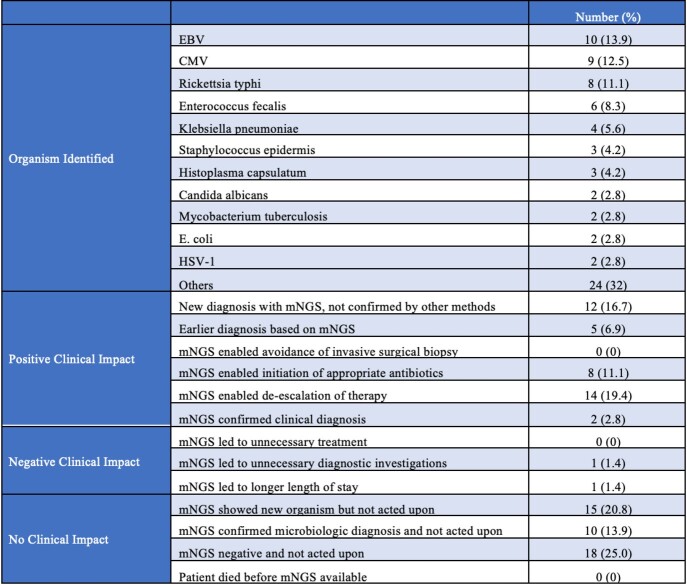
mNGS Test Findings and Impact

**Figure 1:**
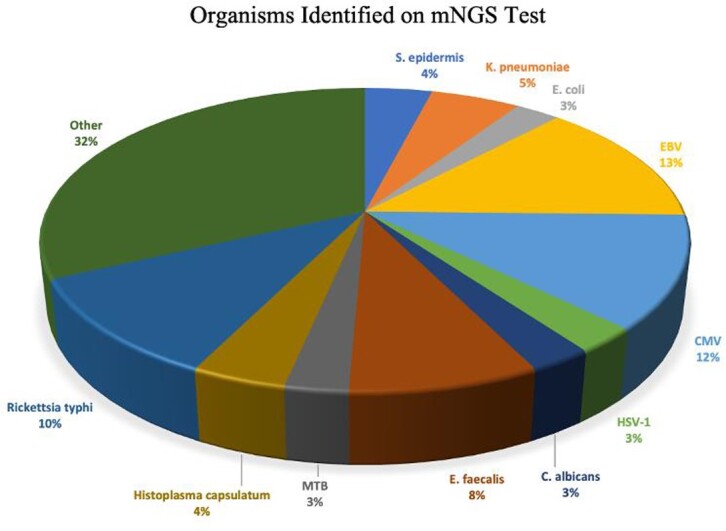
Organisms Identified on mNGS Test

**Conclusion:**

Overall, mNGS was clinically beneficial in a large percentage of patients, with minimal negative impact. Besides age, we did not identify other factors associated with higher likelihood of positive impact. Larger analyses and further studies on the clinical impact of mNGS in different infectious syndromes are needed to better understand this promising tool in the rapid detection of infectious pathogens.

**Table 3:**
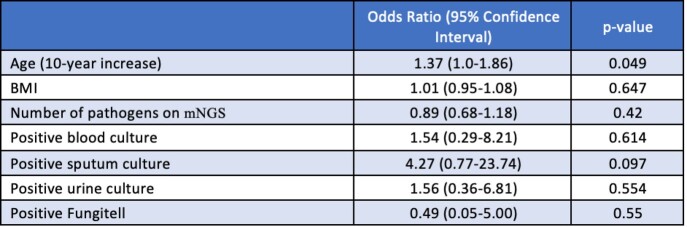
Logistic Regression of Positive Clinical Impact of mNGS

Fungitell® is an FDA-cleared assay that detects 1,3-β-D-glucan in serum

**Figure 2:**
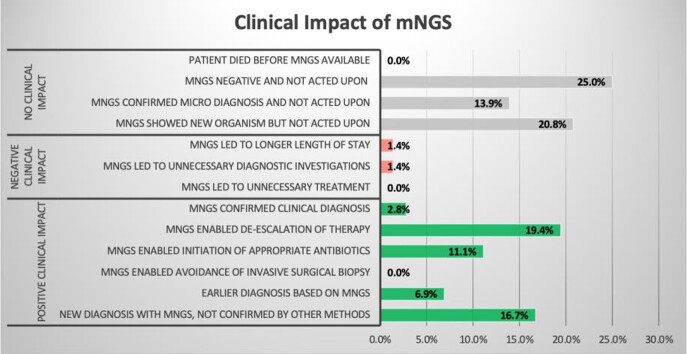
Clinical Impact of mNGS

Clinical impact based on previously published criteria by Hogan et al.

**Disclosures:**

**All Authors**: No reported disclosures.

**Table 2:** mNGS Test Findings and Impact

Abbreviations: Epstein–Barr Virus (EBV); Cytomegalovirus (CMV); Escherichia coli (E. coli); Herpes Simplex Virus-1 (HSV-1)

**Figure 1:** Organisms Identified on mNGS Test

Abbreviations: Staphylococcus epidermis (S. epidermis); Klebsiella pneumoniae (K. pneumoniae); Escherichia coli (E. coli); Epstein–Barr Virus (EBV); Cytomegalovirus (CMV); Herpes Simplex Virus-1 (HSV-1); Candida albicans (C. albicans); Enterococcus faecalis (E. faecalis); Mycobacterium tuberculosis (MTB)

